# Joint Modeling of Transitional Patterns of Alzheimer's Disease

**DOI:** 10.1371/journal.pone.0075487

**Published:** 2013-09-20

**Authors:** Wei Liu, Bo Zhang, Zhiwei Zhang, Xiao-Hua Zhou

**Affiliations:** 1 Department of Mathematics, Harbin Institute of Technology, Harbin, P.R. China; 2 Biostatistics Core, School of Biological and Population Health Sciences, College of Public Health and Human Sciences, Oregon State University, Corvallis, Oregon, United States of America; 3 Division of Biostatistics, Office of Surveillance and Biometrics, Center for Devices and Radiological Health, Food and Drug Administration, Silver Spring, Maryland, United States of America; 4 Department of Biostatistics, School of Public Health and Community Medicine, University of Washington, HSR&D Center of Excellence, U.S. Department of Veterans Affairs Puget Sound Health Care System, Seattle, Washington, United States of America; Cleveland Clinic Lerner Research Institute, United States of America

## Abstract

While the experimental Alzheimer's drugs recently developed by pharmaceutical companies failed to stop the progression of Alzheimer's disease, clinicians strive to seek clues on how the patients would be when they visit back next year, based upon the patients' current clinical and neuropathologic diagnosis results. This is related to how to precisely identify the transitional patterns of Alzheimer's disease. Due to the complexities of the diagnosis of Alzheimer's disease, the condition of the disease is usually characterized by multiple clinical and neuropathologic measurements, including Clinical Dementia Rating (CDRGLOB), Mini-Mental State Examination (MMSE), a score derived from the clinician judgement on neuropsychological tests (COGSTAT), and Functional Activities Questionnaire (FAQ). In this research article, we investigate a class of novel joint random-effects transition models that are used to simultaneously analyze the transitional patterns of multiple primary measurements of Alzheimer's disease and, at the same time, account for the association between the measurements. The proposed methodology can avoid the bias introduced by ignoring the correlation between primary measurements and can predict subject-specific transitional patterns.

## Introduction

Alzheimer's disease is the most common type of dementia with the loss of brain function, which affects mental processes including attention, memory, producing and understanding language, learning, reasoning, problem solving, and decision making. Patients with Alzheimer's disease often are 65 years old or older, but there have been cases of early onset Alzheimer's (diagnosed before the age of 65) [1]. Usually, symptoms of Alzheimer's disease develop gradually and get worse as the disease progresses, and eventually lead to death. Currently, there are no clinical treatments that can cure Alzheimer's disease or reverse its progression, although the safety and efficacy of more than 400 pharmaceutical treatments have been or are being investigated worldwide (approximately a quarter of these compounds are in Phase III trials). According to Alzheimer's Association, 5.4 million Americans lived with Alzheimer's disease in 2012, and the disease is now the 6th leading cause of death in the United States [2]. In this research article, we consider the Uniform Data Set from the Alzheimer's Disease Centers program established by the National Institute on Aging (NIA) [3], [4]. The Alzheimer's Disease Centers program (U.S. National Institutes of Health Grant U01 AG016976) aims at characterizing individuals with mild Alzheimer's disease and cognitive impairment in comparison with nondemented aging. Currently more than 29 Alzheimer Disease Centers are funded to collect demographic, behavioral status, cognitive testing, and clinical diagnoses information on participants. In all Alzheimer Disease Centers, the data in Uniform Data Set are obtained longitudinally in a uniform manner from participants, using standard methods and uniform diagnostic criteria to evaluate subjects. The data are then integrated in the National Alzheimer's Coordinating Center to support sharing and collaborative research.

In numerous clinical and epidemiologic studies on chronic diseases, the scientific interest lies in understanding the longitudinal transitions of primary disease measurements, and in identifying factors that govern transition probabilities. For Alzheimer's disease, the transition patterns contain important information on disease evolution and prognosis. Alzheimer's disease cannot be accurately diagnosed and described by a single measurement; clinicians need to synthesize information from various sources, including features of symptom onset and course, evaluation of mental status, and performance measurements from neurologic and neuropsychological examinations. Therefore, a major challenge in characterizing transitional patterns of Alzheimer's disease is that the disease status is represented by not one but multiple measurements. If the status of Alzheimer's disease could be ascertained by one clinical measurement, then the existing statistical methodologies for univariate transition models would be adequate for the present application. Various authors have proposed models for characterizing transition patterns with univariate longitudinal data: Zeger and Qaqish [5] and Wong [6] proposed Markov regression models and developed estimators based on composite likelihood functions; Albert [7] proposed a transitional model for longitudinal binary data subject to nonignorable missingness and derived an EM algorithm for parameter estimation; Lee and Daniels [8] proposed marginalized transition models for longitudinal binary data and developed Fisher-scoring algorithms for estimation. Albert and Follmann [9] developed a shared random-effects transition model that linked the propensity of transition between states to the probability of an intermittent missed observation or dropout. These methods can be used to analyze each disease measurement separately, but they are not able to account for both the association between different disease measurements and subject-specific transitional patterns.

This research article proposes a class of innovative joint random-effects transition models for elucidating the transitional patterns of multiple longitudinal measurements that are closely related to Alzheimer's disease. Multivariate transition models had been discussed in three recent articles for binary longitudinal data. A generalized estimating equation method for joint transitional models of multivariate longitudinal binary data was proposed by Zeng and Cook [10]. This approach allows modeling of covariate effects on marginal transition probabilities, as well as the association parameters. Ilk and Daniels [11] formulated marginalized random-effects models to accommodate multivariate longitudinal binary data, and Lee et al. [12] later extended these models by using a new covariance matrix with a Kronecker decomposition. All of these methods are focused on the marginal transition patterns of multivariate binary data. In the Alzheimer's disease data, several primary measurements are multi-categorical, and investigators are more interested in subject-specific prognosis than population-averaged prognosis. The joint random-effects transition models proposed in this article involve random effects in each submodel for an individual disease measurement, thereby accounting for the heterogeneity between subjects. Meanwhile, a correlation between disease measurements is induced by the multivariate distribution of the random effects across submodels. In addition, each univariate submodel in the proposed joint random-effects transition models can be flexible enough to accommodate various types of measurements (e.g., Gaussian, multi-categorical, count, etc.). Relative to the existing literature, the benefits of the joint random-effects transition models are multifold: (i) comparing with the modeling of single process in [5], [6], [7], they allow flexible correlation among multiple measurements for a disease; (ii) comparing with the marginal models in [8], [10], [11], they offer insights on patient-specific transitional patterns of disease measurements over time; (iii) comparing with the models for binary data in [10], [11], they offer flexible submodels for fitting the data with various types; and (iv) they can identify common and uncommon covariates that govern the transitional probabilities of each disease outcome. The joint random-effects transition models can help clinicians predict a patient's Alzheimer disease status over time, based on the patient's current status and other genetic or sociodemographic factors.

## Methods

### The National Alzheimer's Coordinating Center: Uniform Data Set

The National Alzheimer's Coordinating Center is responsible for developing and maintaining a database of participant information collected from the 34 past and present Alzheimer's Disease Centers. The NIA organized the Alzheimer's Disease Centers Clinical Task Force and defined a standardized Uniform Data Set. Alzheimer's Disease Centers provide researchers a standard set of assessment procedures to characterize mild Alzheimer disease and mild cognitive impairment in comparison with nondemented controls. Our analysis is based on a data set concerning the status of Alzheimer's disease at five to six annual clinic visits for 746 subjects. The status of Alzheimer's disease for each subject was mainly characterized by four primary measurements: Clinical Dementia Rating, Mini-Mental State Examination, Composite Cognitive Score, and Functional Activities Questionnaire. At each visit, clinicians assessed the overall dementia level of the subject in term of Clinical Dementia Rating, a numeric scale used to quantify the severity of dementia [13], [14]. We denote this first measurement as “CDRGLOB”. CDRGLOB is an ordinal variable with five levels (0, 0.5, 1, 2 and 3, indicating normal status without demetia and questionable, mild, moderate, and severe dementia, respectively). The second primary measurement for the evaluation of cognitive state and impairment was obtained from Mini-Mental State Examination (MMSE), which includes orientation to place and time, registration, attention and concentration, recall, language, and visual construction [15]. The integer score (from 0 to 30) for MMSE was dichotomized by the recommended cutoff score of 23 to detect impairment [15]. In addition to MMSE, another score, denoted by “COGSTAT”, was obtained from neuropsychological examinations [16] and used to assess whether or not a subject's cognitive status was normal for the current age. The fourth primary characteristic of Alzheimer's disease is Functional Activities Questionnaire, denoted by “FAQ”, which represents the level of functional activities of the subject in the community (as related to cognitive dysfunction) [17]. The integer score (from 0 to 30) for FAQ was categorized into five ordinal levels: 0, 1, 2, 3, and 4, representing normal, mildly difficult, moderate, serious and complete disability, respectively.

**Figure 1:**
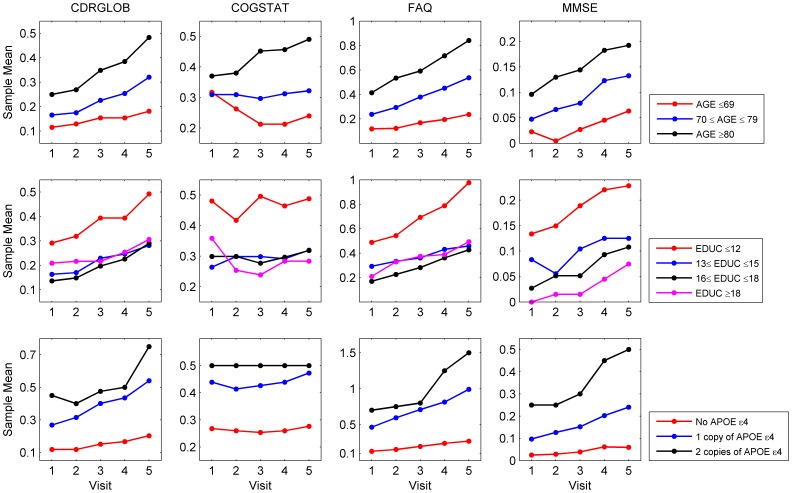
The Alzheimer's disease data: Sample means of four responses in each group presented by age, years of education and the number of copies APOE-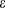 4.

Sloane et al. [18] studied the transitional patterns of Alzheimer's Disease in term of the rating of CDR. However, as indicated earlier, univariate modeling has limitations because it ignores the correlation among different measurements. Using novel joint random-effects transitional we attempt to address three issues in this research article: how the disease condition of the subject would be next year given his or her current diagnosis results in terms of the four primary measurements, how the four primary measurements are correlated through random effects, and how the genetic or sociodemographic factors affect the transitional probabilities. The four primary measurements of Alzheimer's disease are all determined by the participants' underlying condition during a clinical visit. As a result, they are expected to be substantially correlated, and understanding their correlation is a research question of interest. Moreover, patients' age, total years of education, and the number of copies of Apolipoprotein E 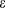
4 allele (APOE-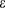
4) are well known risk factors of Alzheimer's disease [19], [20], [21], [22]. It is essential to incorporate those genetic and sociodemographic factors into the joint random-effect transitional models as potential covariates. Hypothesized links between the four primary variables and the potential covariates are shown in Figure 1, in which the means of the four primary variables based on their first five observations are plotted by participant group.

### Joint Transition Models for Bivariate Longitudinal Data

In this section, we initiate the discussion on joint random-effects transitional models by considering bivariate longitudinal data. General transitional models for multivariate longitudinal data (with three or more primary measurements) are discussed in the next section.

Consider a longitudinal study in which 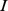 participants are followed prospectively. Longitudinal profiles of the 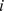
th participant, 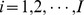, are measured at 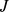 scheduled time point 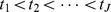, 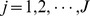, without dropout or intermittent missing values. Assume that a vector of two response variables 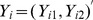 is measured on each participant at each time point, and longitudinal measurements of each response form a stochastic process. The processes for participant 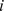 are denoted by 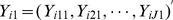 and 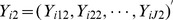, where 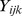 is the response variable at time point 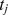 for the 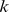
th process, 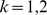. Let 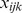 be a 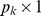 vector of covariates that may be associated with the transition of the 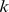
th process at 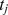. Important covariates in 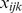 may include a categorical variable denoting treatment group, assessment time 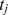 or related time-dependent variables, and risk factors of medical conditions. The history of past 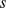 response observations and past 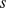 covariates for participant 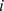 at 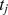 is denoted by 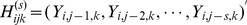. The two processes may be connected through correlated random effects.

We consider several scenarios. In the first scenario, it is assumed that the bivariate longitudinal response 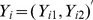 is a mixed-type vector consisting of a Gaussian stochastic process 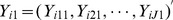 and a binary stochastic process 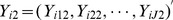. We assume that 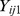 follows a first-order autoregressive correlation model [23], [24] given by
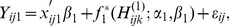
(1)where 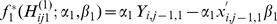. Model (1) implies that the transition pattern for each participant is determined by a linear combination of covariates and past states; that is, participants with identical covariate values and past state values have the same expectation for the current state. This assumption may be unrealistic when there exists heterogeneity in the transition pattern across study participants due to some latent factors. In that case, a more realistic model that counts for the participant-level transition heterogeneity is given by 
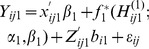
, where 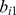 is an unobserved vector of random effects following a mean-zero multivariate normal distribution (independently of the within-participant errors 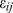), and 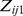 is the random-effects regression vector whose elements are selected from 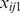 and 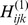. Although we have started by considering a specific form of 
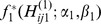
, it is not necessary to assume this particular form. Thus, we now replace 
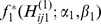
 by 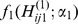 with an arbitrary function (say 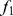) of 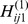 and 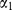 (the collection of all relevant parameters). Then, the random-effects transition model for the continuous process is



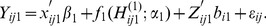
(2)The random-effects component in (2) is flexible enough to include several special cases: transition models with an random intercept correspond to 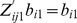, where 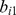 is a scalar random effect; models that characterize between-participant heterogeneity induced by current covariates, with 
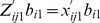
 for a multivariate random effect vector 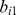 or 
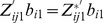
 with 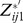 a subset of 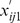; and transition models with between-participant heterogeneity induced by past covariates and past outcome states, with 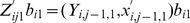 or 
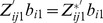
, where 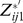 is a subset of 
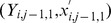
. The random-effects transition model (2) allows a flexible transition pattern across participants but assumes a common conditional mean pattern 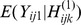. It connects the present state 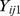 with the past states through both the previous state 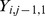 and the common participant-level random effects 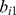.

The random-effects transition model for the binary stochastic process 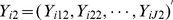, 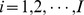, with participant-specific random effects can be constructed in an analogous manner. Suppose each binary realization 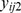 takes value either 1 or 0, indicating the active or inactive status of the process, then the random-effects transition model extended from the marginal first-order two-status Markov chain is
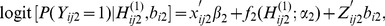
(3)where the regression matrices 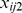, 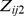, regression coefficients 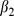, and random effects vector 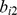 are specified in a way similar to (2). In (3), 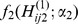 is an arbitrary function of 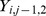 and 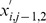, namely, 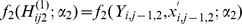. Instead of targeting marginal transition probabilities, (3) is best suited for describing individual transitional patterns based on individual characteristics and random effects.

To formulate a joint random-effects model for the Gaussian and binary mixed-type bivariate longitudinal process, we combine univariate models (2) and (3) through a joint multivariate mean-zero normal distribution for all random effects: 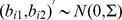. The joint model induces a correlation between the two longitudinal processes through the correlated random effects 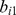 and 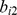. Usually, the correlation between processes cannot be analytically derived. An important special case of the joint model is shared-parameter models where the random effects in (2) and (3) are identical for all study participants (i.e., 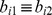). In such models the random effects are treated as shared “parameters”. Note that it is necessary to scale the shared random effects, which means that 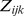 needs to be defined carefully. Shared-parameter models make strong assumptions on the association between outcomes, though it avoids high-dimension integration in maximum likelihood estimation.

In the second important scenario, the bivariate longitudinal outcome 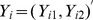 is assumed to be a mixed-type vector with a Gaussian stochastic process 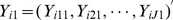 and a count stochastic process 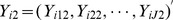. The Gaussian process is assumed to be identical to the one described by (2). The count process 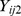, 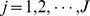, takes values in the set of nonnegative integers. A natural model for 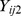 would be a Poisson model with density 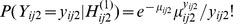, 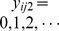, where 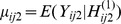 is the intensity or rate parameter for 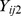. To study the transitional behavior of 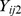, Zeger and Qaqish [5] discussed a first-order Markov model with 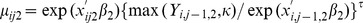, which is equivalent to a log-linear Markov model given by 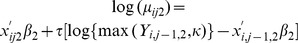. The constant 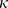 is used to prevent 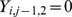 from creating an absorbing state, which would force the future states to be identically 0. When 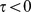 a prior state greater than its expectation decreases the expectation for the current state and consequently 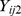 and 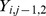 are negatively correlated; whereas when 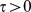 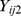 and 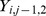 are positively correlated. Random effects can be incorporated into the log-linear Markov model of Zeger and Qaqish [5] to account for participant-level heterogeneity:
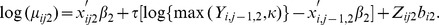



Generally, we consider a log-linear transition model for the count process 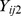 given by
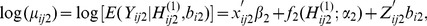
(4)where 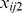, 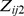, regression coefficients 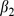, and random effects vector 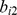 are specified in a way analogous to (2). The general form of 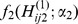, the function that links the current state with the prior state and covariates, includes several special cases: 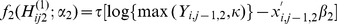 in Zeger and Qaqish [5]; 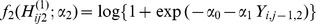 in Wong [6]; or 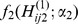 can be 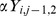, which has limited utility due to the exponentially growing conditional expectation when 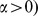. Now, we can combine (2) and (4) into a random-effects joint model by assuming a joint multivariate mean-zero normal distribution for 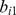 and 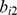: 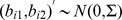.

A joint model for two binary stochastic processes 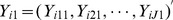 and 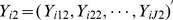 can be specified simply by assuming each behaves as in (3):
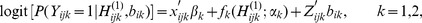
(5)with 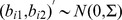. The transition kernels of the two processes are correlated through the matrix 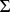.

### Joint Transition Models for Multivariate Longitudinal Data

When there are more than two longitudinal outcomes of different types :(continuous, binary, count, etc.), joint models can be specified within the framework of generalized linear mixed models in a way similar to the case of two outcomes. As before, let 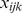 be a 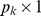 vector and let 
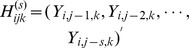
 be the history of past 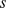 response observations and past 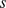 covariates for participant 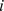 at 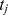 from the 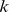 th process. An additional notation 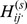 is now introduced to denote the collection of past history for all 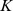 processes: 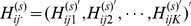. Conditional on random effects 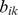 and past history 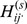, the current state 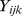 is assumed to be independent across participants and different responses, with a density function of the form 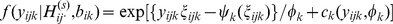, where 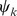, 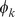 and 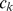 are standard quantities in exponential families. As a standard result for exponential families, we obtain the conditional mean 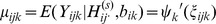 and the conditional variance 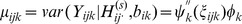. The process-wise transition model is given by
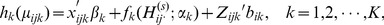
(6)for some link function 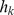. In (6), 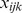, 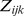, 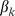, and 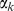 are defined analogously to in (3) and (4). It is worth noting that unlike models (3) and (4), model (6) allows the current state of each individual process to depend on the previous 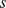 states of all processes involved (i.e., 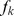 is a function of 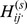, not just 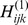). The extra generality translates into added flexibility with respect to the correlation between longitudinal processes. The joint modeling of 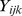
's is finalized by assuming the random effect vectors 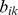 to be correlated as follows:



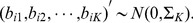
(7)The general formulation (6) and (7) for joint modeling of longitudinal profiles through random-effect transition models provides a general framework for studying complex transition properties for multiple outcomes which are correlated and possibly interact with each other.

### Statistical Inference: Composite Conditional Likelihood

In joint random-effects transition models for multivariate longitudinal data, statistical inference is challenging for several reasons. In (6) and (7), denote by 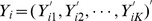 the collection of longitudinal data from the 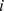
th participant (in the Uniform Data Set of Alzheimer's disease, 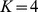). Let 
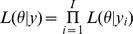
 be the full likelihood based on (6) and (7), where 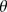 denotes the vector of all unknown parameters (including 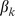
's, 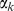
's and the parameters in the variance components). For the 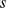
th-order joint transition models, the full likelihood is
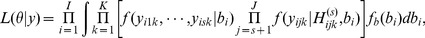
(8)where 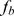 is the mean-zero multivariate normal density of the random effects 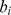. There are two challenges in evaluating the likelihood (8). First, (6) only specifies the conditional distribution 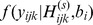 for 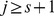, but the conditional distribution of the first 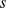 observations 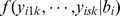 is not determined by (6). As a consequence, the full likelihood (8) is generally not available. Second, the number of stochastic processes, 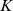, determines the complexity of integration in (8) (i.e., the dimension of integration needed to evaluate 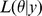). For joint transition models with 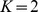, direct evaluation of the full likelihood is possible. However, when 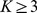, the dimension of integration in evaluating 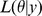 increases dramatically with 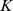, and therefore direct evaluation becomes computationally infeasible.

Here, we construct a well-defined composite conditional likelihood function to overcome the difficulties in evaluating (8). A composite likelihood function is an inference function derived by multiplying a collection of weighted conditional or marginal likelihoods [25], [26]. Composite likelihood is sometimes referred to as pseudo-likelihood or quasi-likelihood, although slight differences exist in some contexts. The advantage of using composite likelihood is that it reduces the integration complexity (relative to the full likelihood), while maintaining the consistency and asymptotic normality of parameter estimates. Composite likelihood can be a composite of conditional likelihood density functions [24], [26], which have been used extensively in univariate transition models (see Diggle et al. [24], Chapter 10) as a way to avoid specification of the marginal distribution of the initial states in a process. In the present context, the conditional likelihood 
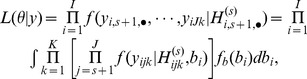
 can be used for inference as an alternative to the full likelihood (8). Here, 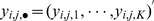 and 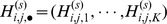. Although this conditional likelihood avoids specifying the marginal distributions of the initial 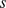 states, it still has the same dimension of integration as the full likelihood (8). To deal with the latter problem, we adopt the pairwise likelihood approach of Fieuws and Verbeke [27] and Faes et al. [28], who proposed a composite marginal likelihood constructed from the marginal likelihood functions based on outcome pairs. The foregoing discussion leads to the following composite conditional likelihood:
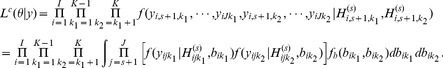
(9)


The composite conditional likelihood (9) focuses on the pairwise conditional likelihood, which avoids high-dimensional integration and specification of the distribution of the first 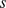 states. Instead of evaluating the high-dimensional integral in the full likelihood (8), only 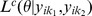 needs to be evaluated, thereby reducing the dimension of integration. In addition, the pairwise conditional likelihood 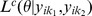 does not require the specification of the joint distribution of the first 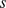 states of the processes.

Inference on the unknown parameters 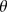 can be based on existing asymptotic results for composite likelihoods [25], [26], [29]. The maximum composite likelihood estimator 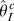 can be found by solving the composite score function 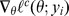, where 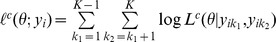.

**Remark 1:** *Under regularity conditions, the composite maximum likelihood estimator 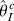 is consistent:*



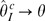



*as* 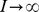. *A central limit theorem for the composite likelihood score statistic holds, which implies that 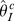 is asymptotically normally distributed:*



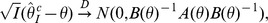



*where*



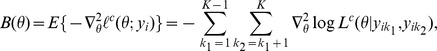



*is the sensitivity matrix,*



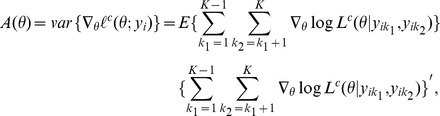



*is the variability matrix, and 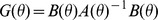 is the Godambe information matrix.*

The proof of Remark 1 is similar to the arguments of [25], [26], [29], and thus is omitted here. For testing the null hypothesis that the unknown parameters equal pre-specified values of interest, the Wald, score, and likelihood ratio test statistics can be extended to the composite conditional likelihood using existing results [26], [29], [30].

The joint modeling approach with the composite conditional likelihood is complicated, possibly involving mixed types of binary or multi-categorical outcomes. Although the composite likelihood asymptotic theories in Remark 1 ensure asymptotic validity, the finite sample performance of the proposed composite likelihood estimators still needs to be thoroughly examined, especially when the outcomes are binary or multi-categorical. We conducted a simulation study to investigate the finite sample performance of 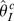. The simulation study was designed to mimic the Alzheimer's disease application. The simulation results, reported in Appendix (see Supporting Information), show that the composite maximum likelihood estimator 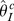 performs well in realistic situations similar to Alzheimer's disease application. In addition, we compared the composite maximum likelihood estimates with estimates derived from a two-stage maximum likelihood approach.

## Results

Two of the four primary measurements of Alzheimer's disease, MMSE and COGSTAT, are dichotomous and can be handled using model (3). The other two measurements, CDRGLOB and FAQ, are both on a five-level ordinal scale. To accommodate the ordinal nature of the latter two measurements, the following random-effects transition model is constructed. For a fixed 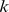, consider an ordinal longitudinal outcome 
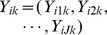
 in which 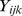, representing the state at time 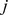, has 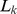 ordinal levels. Covariates and past states are linked with the probability of the event 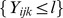 through a random-effects cumulative logit transition model:
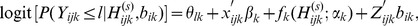
(10)where 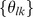 are increasing in 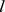. In the linear predictor, the fixed-effects components 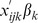, random-effects component 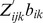, and past states component 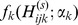 are the same as in (3) and (4), except that each cumulative log odds 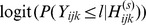 has its own intercept. Each of the four primary measurements 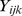 (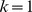 for CDRGLOB; 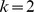 for FAQ; 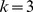 for MMSE; 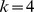 for COGSTAT) can be viewed as an ordinal variable with either five levels or two levels ranging from no sign of symptoms to severe dementia. Therefore, in the following analysis, we used (10) as the random-effects cumulative logit transition model for all four measurements. Precise definitions of ordinal levels have been provided in the Methods section. The vector of covariates 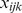 is assumed, for all four measurements, to consist of (1) participant's age at initial visit, (2) years of eduction, (3) clinical visit times, and (4) two dummy variables indicating 1 and 2 copies of APOE-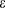
4, which has been suggested as possibly associated with Alzheimer's disease. Finally, the dependence between the four outcomes is characterized by a multivariate normal distribution of 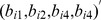, as indicated earlier.

A cautionary note on the usage of (10) is as follows. Investigators have to be very cautious about the specification of 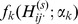 and 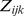. The form of 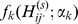 represents the dependence structure of current Alzheimer's disease status on the past, which can include the patients' disease status from only the last clinical visit (i.e., 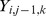) or the last 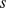 clinical visits (i.e., 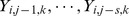). The functional form of 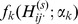 can also vary beyond a linear or additive form. These two aspects have direct impacts on the specification of the random-effects covariate vector 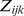, which may consist of any fixed effects in 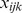 or past disease statues 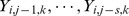. A large number of covariates in 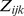 will induce high dimensionality in the normal distribution of random effects, which may not be necessary. In summary, it is important to specify both 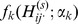 and 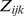 carefully. In our analysis, we first included the last two clinical visits of each patient in 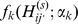 in a linear fashion, in addition to the aforementioned four covariates. Each components of 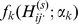 and 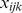 was considered as a possible component of 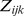, using likelihood ratio tests based on composite likelihood to eliminate unnecessary random effects. This procedure eventually led to the following first-order transition model with a random effect for 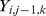 only:
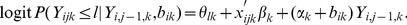
(11)


Our statistical analysis was based on the composite conditional likelihood derived in the Methods section. The SAS programs for this analysis are available upon request. Table 1 presents parameter estimates and (sandwich) standard errors (for both the regression coefficients and the variance-covariance parameters), together with 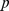-values of Wald tests. The first-order Markov effect is remarkabe for each primary measurement of Alzheimer's disease, with a high level of statistical significance (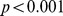) for each 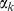, which quantifies the impact of the previous stage disease status 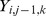. The estimated 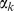 is negative and large in magnitude for each measurement(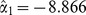; 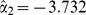; 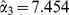; 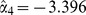 ), indicating that patients with worse Alzheimer's disease conditions in the previous state are more likely to deteriorate. As expected, a patient's age of first-time confirmed diagnosis of Alzheimer's disease plays a significant role for all four measurements. The regression coefficient for age are generally negative and highly significant (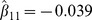, 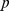-value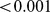; 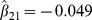, 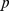-value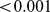; 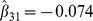, 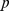-value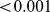; 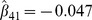, 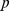-value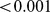), indicating that older patients are more likely to transit into a worse state of Alzheimer's disease (as characterized by the four measurements) at the next clinical visit. This is not only consistent with our previous conjecture, but also corroborates the fact that the cognitive domains were selected for their sensitivity to age-related changes in cognition in neuropsychologic and mental evaluations [31]. Another important factor in the transitional patterns of Alzheimer's disease is the number of copies of APOE-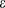
4 allele, again with a high level of statistical significance (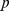-value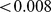 for all 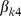
's and 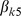
's). The results indicate that Alzheimer's patients with the APOE-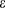
4 allele have higher risks for worse disease symptoms and evaluations than those without the allele in the same current disease situation, and the risk increase is larger for patients with two copies of APOE-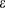
4 than those with only one copy. In Table 1, the effect of education level on the transition of Alzheimer's disease is statistically non-significant for all outcomes but COGSTAT, even though education level is generally considered a prognostic factor that affects the overall cognitive function in Alzheimer's patients. We attempted to investigate the effect of clinical visit times on the transition of Alzheimer's disease, which may be relevant for monitoring annual changes of a patient's condition. However, this effect is not significant in Table 1, suggesting that much of the observed temporal pattern can be explained by the previous disease status. In light of these results, education level and clinical visit time were both removed from the list of covariates in the second part of the analysis (see Table 2).

**Table 1:** Analysis results of the Uniform Data Set in the National Alzheimer's Coordinating Center (four measurements): parameter estimates, sandwich estimators of variance (standard deviations) and covariance, and the 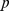-values of Wald tests.

Measurement	Parameter	Estimate	SE	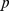 -value
CDRGLOB	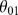 Intercept	5.565	0.668	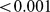
( 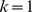 )	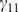 Increment	3.933	0.197	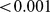
	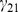 Increment	4.737	0.319	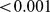
	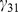 Increment	8.539	0.738	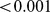
	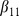 Age	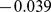	0.007	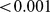
	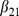 Education	0.031	0.019	0.107
	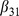 Time	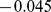	0.038	0.235
	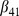 1 APOE	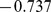	0.111	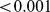
	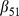 2 APOE	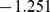	0.261	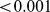
	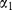 Lag (Coef. of 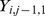 )	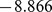	0.359	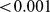
FAQ	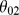 Intercept	7.063	1.099	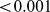
( 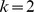 )	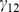 Increment	1.645	0.207	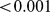
	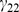 Increment	2.141	0.289	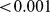
	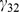 Increment	4.112	0.729	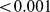
	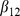 Age	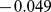	0.011	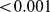
	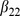 Education	0.033	0.029	0.267
	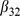 Time	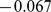	0.052	0.209
	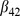 1 APOE	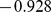	0.175	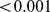
	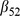 2 APOE	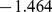	0.501	0.003
	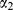 Lag (Coef. of 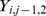 )	−3.732	0.356	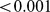
MMSE	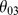 Intercept	10.045	1.667	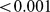
( 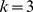 )	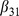 Age	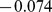	0.016	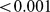
	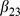 Education	0.024	0.043	0.579
	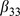 Time	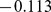	0.072	0.115
	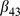 1 APOE	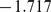	0.255	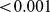
	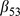 2 APOE	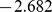	0.485	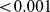
	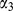 Lag (Coef. of 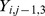 )	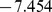	1.301	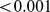
COGSTAT	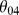 Intercept	5.128	0.655	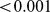
( 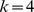 )	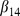 Age	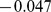	0.008	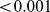
	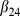 Education	0.055	0.199	0.005
	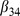 Time	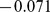	0.033	0.029
	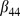 1 APOE	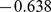	0.109	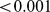
	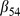 2 APOE	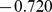	0.271	0.008
	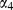 Lag (Coef. of 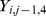 )	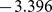	0.441	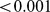
Variance-covariance matrix of random effects				
Variance	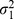 (CDRGLOB)	2.264	0.655	
	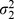 (FAQ)	0.960	0.473	
	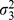 (MMSE)	8.946	6.830	
	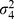 (COGSTAT)	1.021	1.919	
Correlation	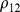 (CDRGLOB, FAQ)	0.508	0.129	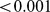
	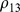 (CDRGLOB, MMSE)	0.576	0.217	0.008
	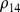 (CDRGLOB, COGSTAT)	0.973	0.492	0.048
	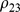 (FAQ, MMSE)	0.754	0.229	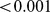
	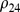 (FAQ, COGSTAT)	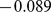	0.693	0.898
	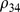 (MMSE, COGSTAT)	0.211	0.917	0.818

(The increment parameters are defined as: 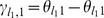, 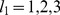; 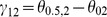; 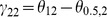; 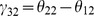).

**Table 2:** Analysis results of the Uniform Data Set in the National Alzheimer's Coordinating Center (three measurements): parameter estimates, sandwich estimators of variance (standard deviations) and covariance, and the 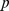-values of Wald tests.

Response	Parameter	Estimate	SE	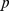 -value
CDRGLOB	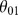 Intercept	5.565	0.517	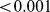
( 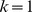 )	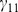 Increment	3.932	0.202	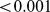
	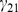 Increment	4.737	0.325	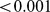
	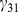 Increment	8.539	0.744	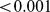
	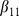 Age	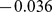	0.007	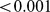
	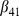 1 APOE	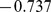	0.109	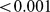
	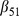 2 APOE	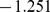	0.251	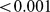
	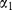 Lag (Coef. of 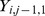 )	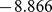	0.370	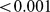
FAQ	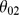 Intercept	7.063	0.858	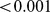
( 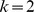 )	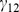 Increment	1.645	0.199	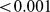
	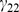 Increment	2.141	0.279	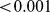
	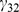 Increment	4.112	0.646	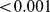
	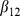 Age	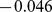	0.011	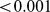
	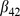 1 APOE	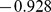	0.174	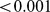
	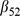 2 APOE	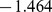	0.466	0.002
	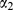 Lag (Coef. of 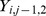 )	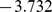	0.326	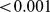
MMSE	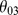 Intercept	10.045	1.213	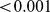
( 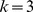 )	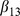 Age	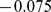	0.015	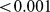
	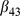 1 APOE	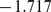	0.235	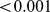
	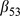 2 APOE	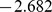	0.459	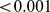
	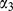 Lag (Coef. of 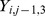 )	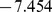	0.849	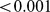
Variance-covariance matrix of random effects				
Variance	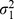 (CDRGLOB)	2.264	0.667	
	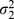 (FAQ)	0.960	0.382	
	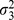 (MMSE)	8.946	3.690	
Correlation	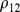 (CDRGLOB, FAQ)	0.508	0.131	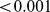
	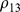 (CDRGLOB, MMSE)	0.576	0.227	0.011
	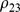 (FAQ, MMSE)	0.754	0.239	0.001

(The increment parameters are defined as: 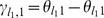, 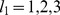; 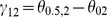; 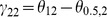; 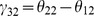).

The lower portion of Table 1 presents estimates of the variance-covariance parameters, which have twofold implications. The correlation estimates of random effects characterize the dependence between four primary measurements of Alzheimer's disease. The significant positive correlations exist between the transitional probabilities of CDRGLOB, FAQ and MMSE (in Table 1, 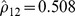, 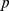-value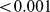; 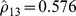, 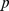-value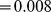; 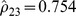, 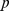-value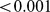). The correlation coefficients related to COGSTAT were all estimated with insignificant or nearly insignificant 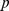-values (in Table 1, 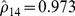, 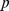-value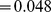; 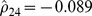, 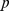-value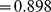; 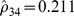, 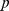-value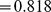). This indicates, when investigating the transitional patterns of Alzheimer's disease, the transition of COGSTAT is not correlated with other three measurements. We therefore excluded COGSTAT from the joint random-effects transition model in the second part of analysis (see Table 2), although this conclusion needs further validation and might be limited to current study cohort. Table 2 reports the results of the second part of refined analysis, which was done by fitting a joint random-effect transition model for CDRGLOB, FAQ and MMSE without patients' clinical visit times and years of education. Comparing with Table 1, the changes in parameter estimates are negligible; some standard errors have shifted in a small amount; and all parameter estimates are highly significant.

Tables 3, 4, 5 and 6 present the matrices of estimated transition probabilities for the four primary measurements, for Alzheimer's patients who are 55 or 85 years old, carrying no, one, or two APOE-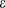
4 alleles, with subject-specific random effects set to zero (corresponding to the median of the population). The results in these tables confirm our previous observations on the effects of age and APOE-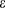
4 allele number on the transitional patterns of Alzheimer's patients. Almost all (more than 99.6%) of the patients who are classified as severe dementia by CDRGLOB will stay that way in the next year's classification. The patients who are classified as in normal condition without dementia will be diagnosed to have Alzheimer's disease in the next year with a probability ranging from 2.6% (Age = 55, no APOE-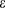
4 allele) to 21.1% (Age = 85, two APOE-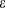
4 alleles), depending on the patient's age and APOE-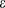
4 allele number. The transition patterns among patients with very mild (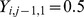) and mild (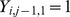) dementia are diverse. The subgroup at age 55 with no APOE-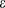
4 allele regresses from very mild to normal condition with a probability of 30.8%, while the subgroup at age 85 with two APOE-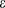
4 alleles regresses with a probability of only 4.2%. The transition probabilities from moderate to severe dementia as measured by CDRGLOB range from 4.3% (Age = 55, no APOE-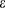
4 allele) to 31.1% (Age = 85, two APOE-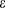
4 alleles). The transitional patterns of functional activities evaluated by FAQ are similar to those for CDRGLOB among normal patients (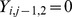) and completely disable patients, but substantially different for patients with mildly difficult (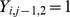), moderate (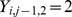), and serious (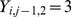) disability. For example, the subgroup at age 55 with no APOE-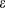
4 allele regresses from mildly difficult functional ability to normal condition with an amazing probability of 69.4%, while patients at age 85 with two APOE-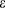
4 alleles regress with a probability of only 11.8%. The transition probabilities for MMSE, shown in Table 5, depend heavily on age and APOE-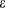
4 allele number, with deterioration probabilities ranging from 0.3% to 27.4% and regression probabilities ranging from 0.2% to 22.0%. The transition probabilities for COGSTAT, shown in Table 6, are further stratified by two levels of education: 12 years and 20 years. There appears to be a small effect of education level on the transitional patterns, but the largest effects are still due to age and APOE-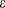
4 allele number. One of the benefits of our joint modeling approach is that the subject-specific random effects can be used for prediction. Once empirical Bayes estimates of the random effects are obtained for a given patient, the transition matrices in Tables 3–6 can be reproduced for this particular patient. This information can help clinicians predict future disease status of this patient.

**Table 3:** The estimated transition probability matrix of clinical dementia rating (CDRGLOB) in Alzheimer's disease patients at the age of 55 or 85 and carrying no, one, or two APOE-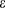 4 alleles.

			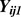				
Age	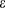 4 alleles	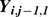	0	0.5	1	2	3
55	0	0	0.9740	0.0254	0.0005	0.0000	0.0000
	1	0	0.9473	0.0517	0.0011	0.0000	0.0000
	2	0	0.9148	0.0833	0.0018	0.0000	0.0000
85	0	0	0.9287	0.0698	0.0015	0.0000	0.0000
	1	0	0.8618	0.1351	0.0031	0.0000	0.0000
	2	0	0.7885	0.2062	0.0052	0.0000	0.0000
55	0	0.5	0.3084	0.6495	0.0417	0.0004	0.0000
	1	0.5	0.1758	0.7400	0.0833	0.0008	0.0000
	2	0.5	0.1132	0.7537	0.1318	0.0013	0.0000
85	0	0.5	0.1340	0.7536	0.1113	0.0011	0.0000
	1	0.5	0.0689	0.7218	0.2070	0.0023	0.0000
	2	0.5	0.0424	0.6509	0.3028	0.0039	0.0000
55	0	1	0.0053	0.2075	0.7558	0.0314	0.0000
	1	1	0.0025	0.1120	0.8220	0.0635	0.0000
	2	1	0.0015	0.0703	0.8264	0.1018	0.0000
85	0	1	0.0018	0.0839	0.8288	0.0854	0.0000
	1	1	0.0009	0.0421	0.7937	0.1633	0.0000
	2	1	0.0005	0.0256	0.7278	0.2460	0.0001
55	0	2	0.0000	0.0000	0.0043	0.9526	0.0430
	1	2	0.0000	0.0000	0.0021	0.9120	0.0859
	2	2	0.0000	0.0000	0.0012	0.8630	0.1358
85	0	2	0.0000	0.0000	0.0015	0.8838	0.1147
	1	2	0.0000	0.0000	0.0007	0.7862	0.2131
	2	2	0.0000	0.0000	0.0004	0.6879	0.3116
55	0	3	0.0000	0.0000	0.0000	0.0031	0.9969
	1	3	0.0000	0.0000	0.0000	0.0015	0.9985
	2	3	0.0000	0.0000	0.0000	0.0009	0.9991
85	0	3	0.0000	0.0000	0.0000	0.0011	0.9989
	1	3	0.0000	0.0000	0.0000	0.0005	0.9995
	2	3	0.0000	0.0000	0.0000	0.0003	0.9997

**Table 4:** The estimated transition probability matrix of functional activities (FAQ) in Alzheimer's disease patients at the age of 55 or 85 and carrying no, one, or two APOE-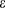 4 alleles.

			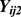				
Age	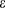 4 alleles	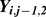	0	1	2	3	4
55	0	0	0.9896	0.0084	0.0018	0.0002	0.0000
	1	0	0.9740	0.0209	0.0045	0.0006	0.0000
	2	0	0.9564	0.0349	0.0077	0.0010	0.0000
85	0	0	0.9601	0.0319	0.0070	0.0009	0.0000
	1	0	0.9049	0.0752	0.0175	0.0023	0.0000
	2	0	0.8478	0.1187	0.0295	0.0040	0.0001
55	0	1	0.6939	0.2276	0.0686	0.0097	0.0002
	1	1	0.4727	0.3501	0.1525	0.0243	0.0004
	2	1	0.3441	0.3869	0.2276	0.0408	0.0007
85	0	1	0.3655	0.3834	0.2131	0.0373	0.0006
	1	1	0.1855	0.3557	0.3681	0.0890	0.0016
	2	1	0.1176	0.2908	0.4461	0.1427	0.0028
55	0	2	0.0515	0.1679	0.4857	0.2881	0.0068
	1	2	0.0210	0.0790	0.3860	0.4970	0.0170
	2	2	0.0124	0.0487	0.2951	0.6151	0.0287
85	0	2	0.0136	0.0531	0.3113	0.5958	0.0262
	1	2	0.0054	0.0220	0.1662	0.7425	0.0638
	2	2	0.0032	0.0131	0.1070	0.7724	0.1043
55	0	3	0.0013	0.0054	0.0475	0.7234	0.2224
	1	3	0.0005	0.0021	0.0195	0.5581	0.4198
	2	3	0.0003	0.0013	0.0115	0.4340	0.5529
85	0	3	0.0003	0.0014	0.0126	0.4560	0.5296
	1	3	0.0001	0.0005	0.0050	0.2542	0.7401
	2	3	0.0001	0.0003	0.0030	0.1671	0.8296
55	0	4	0.0000	0.0001	0.0012	0.0758	0.9228
	0	4	0.0000	0.0001	0.0005	0.0315	0.9680
	0	4	0.0000	0.0000	0.0003	0.0187	0.9810
85	0	4	0.0000	0.0000	0.0003	0.0205	0.9792
	0	4	0.0000	0.0000	0.0001	0.0082	0.9917
	0	4	0.0000	0.0000	0.0001	0.0048	0.9951

**Table 5:** The estimated transition probability matrix of binary cognitive status measured by MMSE in Alzheimer's disease patients at the age of 55 or 85 and carrying no, one, or two APOE-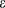 4 alleles.

			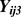	
Age	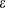 4 alleles	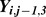	0	1
55	0	0	0.9973	0.0027
	1	0	0.9852	0.0148
	2	0	0.9620	0.0380
85	0	0	0.9749	0.0251
	1	0	0.8745	0.1255
	2	0	0.7264	0.2736
55	0	1	0.1764	0.8236
	1	1	0.0370	0.9630
	2	1	0.0144	0.9856
85	0	1	0.0220	0.9780
	1	1	0.0040	0.9960
	2	1	0.0015	0.9985

**Table 6:** The estimated transition probability matrix of binary cognitive status measured by COGSTAT in Alzheimer's disease patients at the age of 55 or 85, having 12 or 20 years of eduction, and carrying no, one, or two APOE-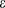 4 alleles.

				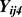	
Education	Age	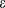 4 alleles	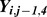	0	1
12	55	0	0	0.9464	0.0536
		1	0	0.9036	0.0964
		2	0	0.8958	0.1042
	85	0	0	0.8311	0.1689
		1	0	0.7232	0.2768
		2	0	0.7055	0.2945
	55	0	1	0.4312	0.5688
		1	1	0.2870	0.7130
		2	1	0.2696	0.7304
	85	0	1	0.1744	0.8256
		1	1	0.1009	0.8991
		2	1	0.0932	0.9068
20	55	0	0	0.9647	0.0353
		1	0	0.9354	0.0646
		2	0	0.9300	0.0700
	85	0	0	0.8828	0.1162
		1	0	0.8015	0.1985
		2	0	0.7873	0.2127
	55	0	1	0.5395	0.4605
		1	1	0.3835	0.6165
		2	1	0.3632	0.6368
	85	0	1	0.2461	0.7539
		1	1	0.1477	0.8523
		2	1	0.1371	0.8629

## Discussion

A new study suggests that the number of people diagnosed with Alzheimer's disease in the United States will triple over the next 40 years [32]. In 2010, 4.7 million people had the disease. By 2050, researchers predict that the number will grow to 13.8 million [32]. Even with this intimating number of prediction, the cause and progression of Alzheimer's disease are still not well understood. Without an effective treatment available for Alzheimer's disease, it is important to understand the progression patterns of the disease. Transitional probabilities based on valid statistical models can help predict disease progression for individual patients. In this research article, we proposed to use joint random-effects multivariate transition models to deliberate the transitional trajectories of Alzheimer's disease in terms of four major measurements. This is the first research article, to our knowledge, that discusses the transition of Alzheimer's disease in a multivariate fashion. The benefits of this methodology includes (i) accounting for the association between measurements, (ii) identifying factors that affect the transition of the disease, and (iii) targeting on the subject-specific transition patterns. The composite conditional likelihood approach makes it feasible to estimate these models.

The joint random-effects transition models have complex structure with several components. There are important and challenging questions, such as how to check the key assumptions. First, statistical tests can be used to compare two nests models. This can help with checking the proportional odds assumption for ordinal responses. Molenberghs and Verbeke [29] described the details on how to conduct likelihood ratio tests for the composite likelihood. Second, model selection criteria based on composite likelihood can be used to compare non-nested models. For instance, Varin and Vidoni [33] proposed an integrated and general information criterion for model selection based on composite likelihood. Gao and Song [34] proposed a composite likelihood version of the Bayes information criterion and established its consistency property for the selection. Both criteria can be adopted to conduct model selection in the joint random-effects transition models, though Gao and Song's method is targeting high-dimensional data. Third, the joint models can be decomposed into marginal distributions (for separate outcomes) and the copula (for the dependence between different outcomes). The marginal models can be checked using existing techniques for a univariate outcome such as residual plots [35]. It appears more difficult to verify assumptions concerning the dependence structure of multiple outcomes (multivariate normal assumption for the random effects). The robustness of joint modeling of multivariate longitudinal profiles on the misspecification of random-effects distribution is an area for future research.

## Supporting Information

File S1 **Appendix.**
(PDF)
