# Supplementary material for: Joint Modeling of Transitional Patterns of Alzheimer's Disease
Source: PLoS One. 2013 Sep 20;8(9):e75487. doi: 10.1371/journal.pone.0075487 (PMC3779177; doi:10.1371/journal.pone.0075487)
Supplement: File S1 — Appendix. (PDF) [file pone.0075487.s001.pdf]

Joint Modeling of Transitional Patterns of Alzheimer's Disease

Appendix: A Simulation Study

Wei Liu[1], Bo Zhang[2,*], Zhiwei Zhang[3], Xiao-Hua Zhou[4]

[1]Department of Mathematics, Harbin Institute of Technology, Harbin, P.R. China
[2]Biostatistics Core, School of Biological and Population Health Sciences, College of Public Health and Human Sciences, Oregon State University, Corvallis, Oregon, U.S.A.
[3]Division of Biostatistics, Office of Surveillance and Biometrics, Center for Devices and Radiological Health, Food and Drug Administration, Silver Spring, Maryland, U.S.A.
[4]Department of Biostatistics, School of Public Health and Community Medicine, University of Washington, HSR&D Center of Excellence, U.S. Department of Veterans Affairs Puget Sound Health Care System, Seattle, Washington, U.S.A.
$*$ E-mail: *Bo.Zhang@oregonstate.edu*

In the research article, a composite conditional likelihood approach is proposed for the joint random-effects transition models. Although the composite conditional likelihood approach can provide an asymptotically consistent estimator (see Remark 1 in the article), it is still necessary to further investigate the finite sample performance of the estimator in realistic situations similar to Alzheimer's disease application. This appendix reports the numerical results from a simulation study that was conducted to evaluate the finite sample performance of the composite conditional likelihood estimator in the joint random-effects transition models. We adopted the joint random-effects transition model that was used for the National Alzheimer's Coordinating Center Alzheimer's disease Uniform Data Set in Table 2 in the article. Two multi-categorical sub-models (with 5-level ordinal categories) for CDRGLOB and FAQ, as well as one binary sub-model for MMSE, were included. The corresponding estimated parameter values obtained in Table 2 were consequently selected as the true parameter values in the simulation study (see Table S.1 for true parameter values), except that the effects of covariates were merged into the intercepts of the sub-models so that we can focus on transitional effects (the effect of Age was replaced by the average of the ages of the study participates; the effect of APOE-ε4 was

replaced by the class of two alleles). Throughout the simulation study, it was assumed that there were 746 subjects as in the Alzheimer's disease Uniform Data Set, and the simulation data were generated from 8 longitudinal clinical visits of each subject. In the simulation study, the initial data from patients' first time clinical visits were prerequisites. From the initial data, we can generate the subsequent observations. Our strategy was that, for each of the 200 simulation data sets, we took one bootstrap sample (one random sample with replacement) of the 746 first-visit values in the Alzheimer's disease Uniform Data Set as the collection of our initial observations for this simulation data set. In the simulation study, we also considered a two-stage approach for estimating the parameters. In this approach, an appropriate univariate random-effects transition model as stated in (11) in the article was fit for each disease measurement. Then, in the second step, the composite conditional likelihood (9) in the article was used to estimate correlation coefficients, with other unknown parameters substituted by their estimates from the first step. Table S.1 in this appendix shows the true parameter values in the simulation study, as well as their estimation biases, standard deviations, and root mean square errors (the square root of mean square errors) given by the composite conditional likelihood and the two-stage inference approach. The results show that the composite conditional likelihood produces reasonably unbiased estimates for the unknown parameters. It outperforms the two-stage approach in estimating the parameters related to the variance-covariance components, especially for the correlation coefficients and the random-effects variances regarding the binary outcome variable. The root mean square errors of ρ_{12}, ρ_{13}, and ρ_{23} given by the composite conditional likelihood are 0.108, 0.081, and 0.076, respectively, whereas they are 0.190, 0.218, and 0.103 in the two-stage approach. The two-stage approach gives 10.539 as the root mean square errors of σ_3 (the variance of the random effect of the binary outcome variable), comparing 0.122 given by the composite conditional likelihood. Both methods perform well in estimating fixed effects. The indication of this simulation study is that, when the investigators are interested in both subject-specific transitional patterns and between-measurement correlations, the direct use of composite conditional likelihood may be preferable. This is in fact proposing major motivation for the use of the joint random-effects transitional models. If the investigators are not interested in between-measurement correlations, they could then work with each outcome separately using traditional univariate transition models.

Table S.1. True parameter values for the joint random-effects models in the simulation study, as well as their estimation biases, standard deviations, and root mean squared errors (RMSE) given by the composite conditional likelihood (CCL) and the two-stage approach.

		CCL			Two-Stage Inference		
Parameter	Truth value	Bias	SD	RMSE	Bias	SD	RMSE
θ_{01} Intercept	1.692	0.061	0.067	0.091	0.019	0.052	0.056
γ_{11} Increment	3.932	0.247	0.125	0.276	−0.049	0.119	0.129
γ_{21} Increment	4.737	−0.088	0.142	0.167	−0.143	0.201	0.246
γ_{31} Increment	8.539	−0.356	0.129	0.378	−0.382	0.424	0.569
α_1 Lag (Coef. of $Y_{i,j-1,1}$)	−8.866	−0.409	0.131	0.429	0.074	0.218	0.230
θ_{02} Intercept	2.204	−0.014	0.067	0.068	0.001	0.061	0.061
γ_{12} Increment	1.645	0.010	0.109	0.109	−0.002	0.077	0.077
γ_{22} Increment	2.141	−0.011	0.148	0.148	−0.020	0.138	0.139
γ_{32} Increment	4.112	−0.207	0.173	0.271	−0.101	0.235	0.255
α_2 Lag (Coef. of $Y_{i,j-1,2}$)	−3.732	0.134	0.114	0.176	0.022	0.130	0.131
θ_{03} Intercept	1.776	−0.012	0.049	0.051	0.006	0.057	0.057
α_3 Lag (Coef. of $Y_{i,j-1,3}$)	−7.454	−0.488	0.166	0.515	−0.418	1.261	1.325
σ_1^2 Variance	2.264	−0.334	0.109	0.351	−0.531	0.466	0.705
σ_2^2 Variance	0.960	−0.701	0.086	0.706	−0.133	0.186	0.228
σ_3^2 Variance	8.946	−0.116	0.040	0.122	2.735	10.212	10.539
ρ_{12} Correlation	0.508	0.026	0.105	0.108	0.095	0.165	0.190
ρ_{13} Correlation	0.576	0.001	0.081	0.081	0.052	0.212	0.218
ρ_{23} Correlation	0.754	0.015	0.075	0.076	0.049	0.091	0.103
